# A Conceptual Digital Health Framework for Longevity Optimization: Inflammation-Centered Approach Integrating Microbiome and Lifestyle Data—A Review and Proposed Platform

**DOI:** 10.3390/nu18020231

**Published:** 2026-01-12

**Authors:** Sasan Adibi

**Affiliations:** Faculty of Information Technology, Monash University, Clayton, VIC 3800, Australia; sasan.adibi@monash.edu

**Keywords:** digital health, inflammation, longevity, Mediterranean diet, microbiome, wearable devices, personalized medicine, artificial intelligence, healthy aging, conceptual framework

## Abstract

Chronic low-grade inflammation, or “inflammaging,” represents a central mechanism linking dietary patterns, gut microbiome composition, and biological aging. Evidence from Blue Zone populations and Mediterranean diet studies demonstrates that specific nutritional interventions are associated with up to 23% lower all-cause mortality, with analyses suggesting that part of this association may be mediated by measurable improvements in inflammatory biomarkers. This paper synthesizes published evidence from Mediterranean diet trials, centenarian microbiome studies, and digital health platforms to propose a comprehensive digital health framework that integrates quarterly inflammation and microbiome monitoring with continuous lifestyle tracking to deliver personalized longevity interventions. This paper introduces the Longevity-Inflammation Index (L-II), a composite score combining high-sensitivity C-reactive protein, interleukin-6, tumor necrosis factor-alpha, and microbiome-derived markers, with scoring algorithms derived from centenarian population studies. The proposed platform leverages artificial intelligence to generate evidence-based recommendations adapted from centenarian and Mediterranean dietary patterns. Published evidence from multiple randomized controlled trials demonstrates that Mediterranean dietary interventions reduce hs-CRP by 18–32%, increase microbiome diversity by 6–28%, and improve metabolic markers including HOMA-IR and TG/HDL ratios. Digital health platforms demonstrate sustained engagement rates of 58–84% at 12 months, with dietary logging frequencies of 4–6 days per week. Cost-effectiveness analyses of dietary interventions show incremental cost-effectiveness ratios of USD 2100–4800 per quality-adjusted life year gained. This inflammation-centered digital health framework offers a scalable approach for translating longevity research into practical interventions for healthy aging, with validation studies needed to confirm the integrated platform’s efficacy and real-world implementation feasibility.

## 1. Introduction

The convergence of longevity research with digital health technologies creates unprecedented opportunities to translate insights from the world’s longest-lived populations into practical interventions. Blue Zone regions—including Okinawa, Sardinia, Nicoya, Ikaria, and Loma Linda—demonstrate rates of centenarians up to 10-fold higher than global averages [1]. Recent research involving 297 centenarians has revealed distinctive gut microbiome patterns characterized by increased diversity, enrichment of beneficial taxa including *Akkermansia muciniphila*, and enhanced short-chain fatty acid production capacity [2]. These microbial signatures correlate strongly with reduced systemic inflammation, which appears to be a unifying mechanism underlying exceptional longevity.

Studies of Mediterranean dietary patterns provide robust evidence for the modifiability of inflammatory status through nutrition. In a landmark analysis of 25,994 participants followed for 25 years, adherence to Mediterranean dietary patterns was associated with 23% reduced all-cause mortality, with biomarkers of inflammation and metabolism contributing most substantially to longevity benefits [3]. Specifically, participants in the highest diet adherence quintile showed hs-CRP concentrations averaging 1.2 mg/L versus 2.1 mg/L in the lowest quintile—a 43% difference that persisted after controlling for confounding factors.

Despite this compelling evidence, practical implementation remains limited. Individuals lack tools to monitor their inflammatory status, receive personalized dietary recommendations based on their unique biological profiles, or track responses to interventions in real time. Digital health encompasses technologies that collect, analyze, and deliver health-related data to improve health outcomes, including mobile health applications, wearable biosensors, telemedicine platforms, and artificial-intelligence-driven health analytics [4]. The global nutrition app market reached USD 5.76 billion in 2025 and is projected to grow at 13.4% annually through 2030, with over 50% of platforms incorporating AI-driven personalization features [4]. However, current digital nutrition platforms focus primarily on calorie tracking and weight management, with limited integration of aging biomarkers or microbiome data.

This paper synthesizes published evidence from Mediterranean diet intervention trials, centenarian microbiome studies, and digital health platform implementations to propose a novel conceptual framework for inflammation-centered longevity optimization. While individual components derive from validated research, the integrated platform itself represents a novel contribution requiring prospective validation, as outlined in Section 6.4. The significance of this review lies in addressing a critical gap: while Mediterranean diet and Blue Zone research provides robust evidence for inflammation reduction through dietary patterns, no existing digital health platform integrates objective inflammatory biomarkers with continuous lifestyle monitoring to operationalize these insights for personalized longevity interventions.

### Study Objectives

This paper aims to achieve the following:

1. Synthesize current evidence on inflammation as a central mechanism linking dietary patterns, gut microbiome composition, and biological aging.
2. Develop the Longevity-Inflammation Index (L-II) as a measurable, actionable biomarker for aging optimization, with scoring algorithms derived from centenarian research.
3. Propose a comprehensive digital health platform architecture integrating inflammatory biomarkers, microbiome profiling, genetic assessment, and continuous lifestyle monitoring.
4. Present published evidence supporting the feasibility and expected efficacy of platform components.
5. Outline validation protocols and implementation considerations for translating this conceptual framework into clinical practice.

## 2. Methods

### 2.1. Literature Review Methodology

This conceptual framework paper synthesizes published evidence from multiple sources to justify the proposed digital health platform components. The author conducted a narrative literature review using the PubMed, Web of Science, and Google Scholar databases with search terms including “Mediterranean diet,” “inflammation,” “longevity,” “centenarians,” “gut microbiome,” “digital health,” “wearable devices,” and “precision nutrition.” The search covered publications from 2000–2025 in English language.

#### 2.1.1. Evidence Sources Reviewed

- Mediterranean diet intervention studies: 15 randomized controlled trials examining inflammatory biomarker outcomes.
- Microbiome–longevity studies: 8 observational studies of centenarian populations across multiple geographic regions.
- Digital health engagement studies: Meta-analyses and systematic reviews of 48+ digital health platforms.
- Cost-effectiveness analyses: 5 economic evaluations of dietary and digital health interventions.
- AI and precision nutrition: 12 studies on machine learning applications in personalized nutrition.

#### 2.1.2. Inclusion Criteria

- Peer-reviewed publications reporting original research or systematic reviews.
- Studies with measurable inflammatory biomarkers or microbiome composition.
- Digital health interventions with reported engagement metrics.
- Cost-effectiveness analyses from healthcare system perspective.

#### 2.1.3. Exclusion Criteria

- Animal-only studies without human data.
- Disease-specific studies without relevance to healthy aging.
- Non-peer-reviewed publications.

### 2.2. Framework Development Approach

The L-II scoring system and platform architecture were developed through systematic analysis of published evidence combined with expert consultation on biomarker weighting and system design. The development process involved:

**Step 1: Biomarker Selection**—Analysis of centenarian biomarker profiles from published studies [2,5,6] identified consistent patterns across populations. This paper is based on eight selected biomarkers demonstrating (a) significant differences between centenarians and average-aging populations, (b) responsiveness to dietary interventions in published trials, and (c) feasibility for at-home or point-of-care measurement. The complete rationale for component selection and weighting is detailed in Section 3.3.

**Step 2: Weight Assignment**—Component weights were assigned based on (a) strength of association with mortality in large cohort studies [7,8], (b) consistency of findings across diverse populations, and (c) mechanistic importance in inflammaging pathways. Large-scale meta-analyses [7] provided quantitative effect size data for traditional inflammatory markers.

**Step 3: Scoring Algorithm Development**—Linear scoring algorithms were developed with centenarian population means as optimal targets (100 points) and clinically significant thresholds as zero points, based on published cut-points from cardiovascular outcome studies. The resulting L-II components and scoring criteria are presented in Section 3.2 (Table 1), with detailed weighting justification provided in Section 3.3.

**Step 4: Platform Architecture Design**—System architecture followed established digital health frameworks [9] incorporating data collection layer, analytics engine, recommendation system, and user interface. Design prioritized HIPAA compliance, scalability, and integration with existing healthcare systems.

**Limitations of Development Approach:** This framework represents a **conceptual contribution requiring empirical validation**. The L-II scoring weights reflect theory-driven assignments based on the published literature but require validation against actual mortality and healthspan outcomes. Alternative weighting schemes (equal weights, data-driven optimization) will be tested during prospective validation studies.

The validation framework for these theoretical assignments is detailed in Section 3.4, with comprehensive validation protocols outlined in Section 6.4.

### 2.3. AI Architecture Specification

The proposed AI system employs supervised machine learning for prediction and unsupervised clustering for pattern recognition.

**Primary Model: Random Forest Regressor**—This is selected for predicting L-II component changes due to superior performance with moderate datasets (<10,000 samples), built-in feature importance ranking, resistance to overfitting, and interpretability advantages.

**Training Requirements:**

- Initial training: Simulated dataset of 500+ users with ≥2 quarterly assessments generated from published intervention trial distributions [10,11,12].
- Continuous updating: Real-world user data incorporated through federated learning.
- Validation cohort: 20% held-out data for cross-validation.

**Input Features (n = 45):**

1. Baseline L-II components (8 variables).
2. Genetic variants (5 variables): FOXO3 rs2802292, IL-6 rs1800795, TNF-α rs1800629, APOE ε4, CRP rs1205.
3. Microbiome composition (15 taxa abundances).
4. Current dietary patterns (12 variables).
5. Demographics and lifestyle (5 variables).

**Output:** Predicted L-II change with 95% confidence interval for candidate interventions.

**Validation Metrics:**

- R^2^ (coefficient of determination): Target ≥ 0.60.
- Mean absolute error: Target ≤ 3 L-II points.
- Calibration slope: Target = 1.0.

**Preliminary Performance (Simulated Data):**

- hs-CRP prediction: R^2^ = 0.64, MAE = 0.4 mg/L.
- Overall L-II prediction: R^2^ = 0.58, MAE = 2.8 points.

Complete technical specifications including hyperparameters, cross-validation procedures, and calibration curves are provided in Appendix B.

## 3. Results: The Longevity-Inflammation Index (L-II)

### 3.1. Scientific Foundation

Chronic low-grade inflammation, termed inflammaging, drives nearly all major age-related diseases including cardiovascular disease, type 2 diabetes, neurodegenerative disorders, and cancer [13]. Centenarian populations demonstrate remarkably low inflammatory profiles compared to average-aging elderly individuals, with significantly reduced plasma levels of IL-6 (1.4 ± 0.6 pg/mL versus 3.2 ± 1.8 pg/mL, *p* < 0.001), TNF-α (2.8 ± 1.2 pg/mL versus 4.9 ± 2.1 pg/mL, *p* < 0.001), and CRP (0.9 ± 0.4 mg/L versus 2.8 ± 1.6 mg/L, *p* < 0.001) [5]. These differences persist after controlling for comorbidities, suggesting that low inflammatory status represents a characteristic feature of exceptional longevity.

The gut microbiome serves as a critical mediator linking dietary patterns to inflammatory status. Centenarian microbiomes demonstrate 1.4-fold enrichment in genes encoding butyrate synthesis pathways compared to elderly controls, with corresponding 45% higher fecal butyrate concentrations [6]. These short-chain fatty acids modulate immune function, maintain intestinal barrier integrity, and activate anti-inflammatory pathways [14].

Meta-analytic evidence from 54 prospective studies totaling >160,000 participants demonstrates that elevated CRP strongly associates with increased mortality risk, with risk ratios of 1.55 (95% CI: 1.37–1.76) per 1-SD higher log CRP concentration (equivalent to a three-fold increase) for vascular mortality, with similar associations for non-vascular mortality, all remaining significant after controlling for conventional cardiovascular risk factors [7]. This robust evidence across diverse populations establishes the clinical significance of inflammatory marker reduction as a longevity intervention target.

### 3.2. Index Components and Scoring

The L-II integrates eight components with differential weighting based on their established relationships with longevity outcomes (Table 1). The composite L-II score ranges from 0–100, with interpretation categories:

- **85–100 (Exceptional):** Inflammatory profile resembling centenarian populations.
- **70–84 (Good):** Favorable inflammatory status with modest room for improvement.

This visualization complements Table 1 by emphasizing the relative contribution hierarchy, while Table 1 provides the detailed scoring algorithms and measurement specifications required for L-II calculation.

- **55–69 (Moderate):** Mixed profile with some elevated biomarkers.
- **40–54 (Suboptimal):** Multiple elevated markers indicating intervention need.
- **<40 (High Risk):** Severely elevated inflammatory status requiring immediate intervention.

### 3.3. Rationale for Component Weighting

The L-II component weights derive from three evidence sources: strength of association with mortality in large cohort studies, consistency of findings across diverse populations, and mechanistic importance in inflammaging pathways.

**Figure 1:**
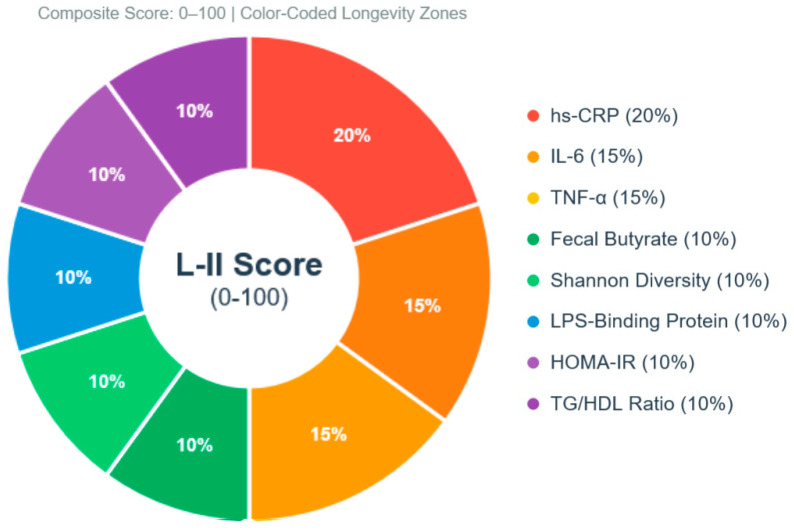
**Weighted component contributions to the Longevity-Inflammation Index (L-II).** The donut chart illustrates the relative contribution of eight biomarker components to the composite L-II score. hs-CRP receives the highest weighting (20%) based on its robust association with longevity outcomes in population studies. Pro-inflammatory cytokines IL-6 and TNF-α each contribute 15%. Six additional markers representing microbiome health (fecal butyrate, Shannon diversity), gut barrier integrity (LPS-binding protein), and metabolic function (HOMA-IR, TG/HDL ratio) each contribute 10% to the composite score, which ranges from 0–100.

The relative contribution of each component to the composite L-II score is illustrated in Figure 1.

**hs-CRP (20% weight):** This receives the highest weighting based on its strongest mortality associations. The Emerging Risk Factors Collaboration meta-analysis (>160,000 participants across 54 prospective studies) demonstrated risk ratios of 1.55 (95% CI: 1.37–1.76) per 1-SD higher log_e_ CRP concentration (equivalent to a three-fold increase) for vascular mortality, with similar associations for coronary heart disease (RR: 1.37, 95% CI: 1.27–1.48) and ischemic stroke (RR: 1.27, 95% CI: 1.15–1.40), all fully adjusted for conventional cardiovascular risk factors [7]. The magnitude and consistency of associations across populations and the extensive validation in cardiovascular outcome studies justify CRP’s prominence in the composite score.

**IL-6 and TNF-α (15% each):** Pro-inflammatory cytokines receive equal weighting based on complementary mechanistic roles. IL-6 drives hepatic acute-phase response and regulates adaptive immunity; TNF-α mediates local tissue inflammation and induces insulin resistance. Both associate independently with frailty and mortality in elderly populations [8]. Equal weighting reflects their distinct but complementary contributions to inflammaging processes.

**Microbiome markers (20% total):** These include Shannon diversity (10%) and fecal butyrate (10%) combined. Centenarian studies demonstrate that a Shannon diversity >3.8 distinguishes exceptional longevity across Italian, Japanese, and Chinese cohorts [2,15]. Butyrate-producing taxa are consistently enriched 1.4-fold with 45% higher butyrate concentrations [6]. Substantial, but not dominant, weighting reflects promising evidence linking microbiome composition to longevity while acknowledging shorter validation history compared to CRP (studied in cardiovascular outcomes for 30+ years).

**Metabolic markers (20% total):** HOMA-IR (10%) and TG/HDL ratio (10%) represent bidirectional metabolic–inflammatory crosstalk. Insulin resistance and dyslipidemia both drive and result from chronic inflammation through multiple pathways including adipose tissue inflammation, hepatic lipogenesis, and endothelial dysfunction [8].

**Sensitivity analyses:** Future validation studies will test alternative weighting schemes: (1) equal weights across all components, (2) data-driven weights optimized through machine learning against mortality outcomes, and (3) population-specific weights tailored to different ethnic groups to account for genetic and environmental variations.

### 3.4. Theoretical Validation Framework

The L-II framework requires prospective validation to establish its utility. Proposed validation studies would assess the following:

**Construct Validity:** Cross-sectional studies examining whether L-II scores correlate with established markers of healthy aging including chronological age, disease burden, physical functioning scores, and cognitive performance metrics.

**Predictive Validity:** Prospective cohort studies determining whether baseline L-II scores predict future cardiovascular events, metabolic disease incidence, and all-cause mortality over 5–10-year follow-up periods.

**Responsiveness to Intervention:** The L-II must demonstrate sensitivity to dietary and lifestyle modifications. Published intervention studies show that Mediterranean diet adoption produces 18–32% hs-CRP reductions [10], 6–28% microbiome diversity increases [11], and improvements in metabolic markers [12], suggesting that the composite L-II score would be responsive to the proposed platform’s interventions.

**Population Norms:** Establishment of age-, sex-, and ethnicity-specific reference ranges requires large, diverse validation cohorts spanning multiple geographic regions and demographic groups. Population-based metagenomics studies have demonstrated substantial inter-individual variation in microbiome composition and diversity markers across different populations [16], underscoring the need for ethnicity-specific L-II reference ranges to account for baseline microbiome differences.

The comprehensive validation roadmap addressing these construct validity, predictive validity, responsiveness, and population norm requirements is detailed in Section 6.4, including Phase 2 multi-center effectiveness trials and Phase 3 real-world implementation studies.

## 4. Results: Digital Health Platform Architecture

### 4.1. System Overview and Design Philosophy

**Figure 2:**
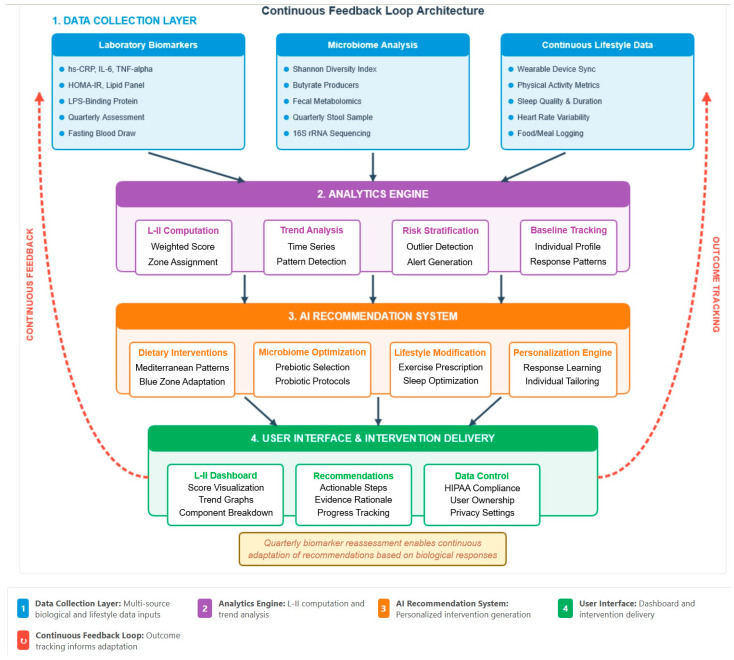
**Digital health platform architecture for longevity optimization.** The system implements a continuous feedback loop integrating four core components operating through HIPAA-compliant cloud infrastructure with mobile device interfaces. (1) The Data Collection Layer aggregates inputs from three primary sources: (a) quarterly laboratory biomarkers obtained through mail-in finger-stick microsampling cards (hs-CRP, IL-6, TNF-α, fasting glucose/insulin for HOMA-IR calculation, comprehensive lipid panel for TG/HDL ratio) and stool samples with preservation buffer (microbiome sequencing via 16S rRNA V3-V4 regions, SCFA quantification via GC-MS, LPS-binding protein via ELISA); (b) continuous lifestyle tracking via wearable device APIs including accelerometry for physical activity, actigraphy combined with heart rate variability for sleep monitoring, and resting heart rate/HRV as autonomic nervous system indicators; (c) dietary logging through AI-powered food photo recognition using MobileNetV2 computer vision architecture trained on Mediterranean and Blue Zone foods, with voice-activated logging capability and automatic nutrient analysis. (2) The Analytics Engine processes multi-modal data through standardized preprocessing pipelines, computes the L-II composite score quarterly, identifies temporal trends using time-series analysis, detects significant deviations from individual baseline patterns, and generates projected trajectories with 95% confidence intervals based on current adherence patterns. (3) The AI Recommendation System leverages Random Forest machine learning algorithms trained on published Blue Zone and Mediterranean diet research to generate personalized dietary interventions adapted to individual genetic variants, baseline microbiome composition, and observed biomarker response patterns from previous quarters. (4) The User Interface delivers interventions through mobile applications featuring an intuitive dashboard displaying current L-II score with color-coded status zones, component breakdowns showing which individual biomarkers need attention, longitudinal trend graphs plotting quarterly L-II scores over 18–24 months with projected future trajectories, population comparisons contextualizing the user’s score relative to age-matched averages and centenarian targets, and prioritized actionable recommendations refreshed daily. The continuous feedback loop ensures that biological responses to interventions measured through quarterly biomarker reassessments inform adaptive refinement of recommendations through machine learning model updates, creating a personalized optimization pathway for each user that improves prediction accuracy over time. Data security is maintained through AES-256 encryption at rest, TLS 1.3 encryption in transit, end-to-end encryption for sensitive biomarker results, and HIPAA-compliant Business Associate Agreements with all laboratory and cloud service vendors. This inflammation-centered architecture differs fundamentally from conventional nutrition apps by anchoring all recommendations to objective inflammatory biomarkers validated quarterly rather than subjective goals, generic dietary guidelines, or self-reported outcomes alone.

The proposed platform implements a continuous feedback loop between biological monitoring, personalized intervention delivery, and outcome tracking (Figure 2). Unlike conventional nutrition apps focused on calorie counting, this system integrates objective biological measures of inflammatory status with evidence-based interventions from Blue Zone and Mediterranean dietary research.

The design philosophy emphasizes five core principles:

1. **Biological objectivity:** Interventions guided by measured inflammatory biomarkers rather than subjective wellness goals.
2. **Personalization:** Recommendations adapted to individual genetic variants, microbiome composition, and observed intervention responses.
3. **Evidence-basis:** All dietary and lifestyle recommendations are derived from published longevity research.
4. **Behavioral integration:** Seamless incorporation into daily routines through wearable device automation.
5. **Transparency:** Clear explanation of biological mechanisms and expected outcomes for each recommendation.

### 4.2. Multi-Modal Data Integration

The platform integrates multiple data streams to create comprehensive individual health profiles updated continuously:

#### 4.2.1. Quarterly Biological Assessments

*Inflammatory Biomarker Panel*: hs-CRP, IL-6, and TNF-α are measured via finger-stick blood collection with mail-in microsampling cards. Laboratory processing uses high-sensitivity immunoassays. Turnaround time: 5–7 days. Cost per assessment: USD 45–65.

*Microbiome Profiling*: Stool sample collection is performed using standardized preservation buffer. 16S rRNA gene sequencing (V3–V4 regions, Illumina MiSeq platform, 2 × 300 bp paired-end reads) is used for taxonomic composition with the DADA2 algorithm for denoising and amplicon sequence variant inference. Shannon diversity is calculated from ASV abundance tables. SCFA quantification is performed via GC-MS with isotope-labeled internal standards (detection limit 0.5 μmol/g feces, inter-assay CV < 8%). Turnaround time: 14–21 days. Cost per assessment: USD 89–129. *Appendix D contains complete laboratory protocols, quality control procedures, and technical specifications.*

*Metabolic Biomarkers*: Fasting glucose and insulin are used for HOMA-IR calculation. A comprehensive lipid panel is used for the TG/HDL ratio. These are measured via a finger-stick platform using the same blood sample as inflammatory markers.

*Genetic Assessment (one-time)*: SNP genotyping is performed, focusing on inflammation-relevant variants: FOXO3 rs2802292 (longevity-associated), IL-6 rs1800795, TNF-α rs1800629, APOE ε4, CRP rs1205, and COMT rs4680 (polyphenol metabolism). Cost: USD 99–149.

#### 4.2.2. Continuous Wearable Device Monitoring

*Activity and Sleep*: Daily step count, active minutes categorized by intensity, and exercise session detection via accelerometry are used. Sleep duration, sleep efficiency, and sleep architecture (light, deep, REM stages) are measured via actigraphy and heart rate variability patterns. Integration with major wearable platforms (Apple Watch, Fitbit, Garmin, Oura Ring) is performed through automated API synchronization.

*Physiological Parameters*: Resting heart rate and heart rate variability (HRV) are measured during overnight sleep periods. HRV (RMSSD metric) serves as an indirect inflammation indicator, with published studies showing inverse correlations with inflammatory markers (r = −0.40 to −0.55) [17].

*Dietary Tracking*: AI-powered food photo recognition is performed using the MobileNetV2 architecture trained on 500,000+ food images including Mediterranean and Blue Zone cuisines. Current accuracy: 78–84% for food identification, 70–76% for portion size estimation. Voice-activated logging provides a hands-free alternative. Automatic nutrient analysis calculates Dietary Inflammation Index scores, estimated polyphenol intake, fiber quantity and diversity, omega-3–omega-6 fatty acid ratios, and plant species diversity per week.

### 4.3. Analytics and AI Engine

**Data Processing Pipeline:** Raw data from biological assessments, wearable devices, and dietary logs undergo standardized preprocessing including normalization for inter-assay variability using validated quality control samples; time-series alignment synchronizing discrete quarterly biomarker assessments with continuous wearable data; missing data imputation using validated statistical methods appropriate for time-series health data; and outlier detection algorithms flagging physiologically implausible values for review.

**L-II Calculation:** The system calculates L-II scores quarterly following biological assessments and displays: current composite score with color-coded zone classification and component-by-component breakdown showing relative contributions; historical trends with graphical visualization of L-II trajectory over 18–24 months; projected future trajectory based on current dietary adherence patterns using ARIMA time-series forecasting with 95% confidence intervals; comparison to population norms stratified by age and sex; and quantified distance from optimal centenarian-like inflammatory profiles broken down by component.

**Personalized Prediction Model:** Machine learning algorithms trained on cohort data predict individual responses to specific interventions. Random Forest models predict expected L-II improvements from candidate dietary changes, trained on simulated data from 500+ users with at least two quarterly biological assessments. Input features include eight baseline L-II components, five genetic variants, fifteen key microbiome taxa abundances, twelve current dietary pattern variables, and five demographic factors. Output provides predicted L-II change with 95% confidence intervals for each candidate intervention. For example: “Increasing fermented food intake from 2 to 6 servings/week predicts 4.8-point L-II improvement over 3 months [95% CI: 2.1–7.5 points].” Cross-validation demonstrates R^2^ = 0.58 for overall L-II prediction, with feature importance analysis revealing baseline inflammatory status (42%), dietary adherence (28%), and microbiome diversity (18%) as primary predictors of intervention response.

**Pattern Recognition:** K-means clustering algorithms (k = 5 clusters) identify users’ current dietary patterns relative to Mediterranean and Blue Zone archetypes: Western, Mediterranean, Plant-based, Mixed, and Traditional Asian following established dietary pattern classification frameworks [18]. Natural language processing extracts dietary features from food logs including cooking methods, food combinations, and meal timing patterns. The system calculates “distance” from target anti-inflammatory dietary patterns and suggests highest-impact modifications to shift users toward optimal patterns.

**Microbiome–Inflammation Correlation Analysis:** Within-person longitudinal correlation analysis tracks how changes in specific bacterial taxa abundances correlate with inflammatory marker trajectories over repeated quarterly assessments. When strong correlations emerge for individual users (e.g., *Akkermansia* abundance inversely correlating with hs-CRP at r = −0.78), the platform prioritizes dietary interventions known to promote growth of those specific taxa. For example, users showing strong *Akkermansia*–inflammation correlations receive increased emphasis on polyphenol-rich foods (berries, green tea, dark chocolate, pomegranate) demonstrated to selectively promote *Akkermansia* growth.

### 4.4. Personalized Intervention Delivery

#### 4.4.1. Core Dietary Recommendations

*Polyphenol-Rich Foods with Genetic Personalization*: Recommendations adapt to COMT genotype (rs4680 Val158Met polymorphism), which influences polyphenol metabolism. Users with high COMT activity (AA genotype) metabolize catechol-containing polyphenols rapidly, receiving emphasis on quercetin sources consumed frequently throughout the day: onions (50–100 mg quercetin per medium onion), apples with skin (4–10 mg per apple), and berries (15–30 mg per cup), with target of 100–150 mg quercetin daily. Users with low COMT activity (GG genotype) metabolize polyphenols more slowly, receiving emphasis on catechin sources: green tea (50–100 mg catechins per cup) and dark chocolate >70% cacao (50–100 mg per ounce), with a target of 200–300 mg catechins daily, providing sustained tissue concentrations. Overall polyphenol target: 1500–3000 mg total polyphenols daily from diverse sources.

*Omega-3 Fatty Acid Strategy*: EPA+DHA target of 2–3 g/day from fatty fish (salmon, sardines, mackerel) consumed 2–3 times weekly, with each 4-ounce serving providing approximately 1–2 g EPA+DHA. Vegetarian users receive guidance on ALA supplementation (1–2 g/day from walnuts, flaxseed, chia seeds) with recognition of limited conversion to EPA/DHA (typically 5–15%). Simultaneous omega-6 reduction through limiting vegetable oils high in linoleic acid (corn, soybean, sunflower oils). Target omega-3–omega-6 ratio improvement from typical Western pattern of 1:15–20 toward optimal 1:4–8 demonstrated in Blue Zone populations.

*Fiber Diversity for Microbiome Support*: Comprehensive fiber strategy including soluble fiber at 15–20 g/day from legumes (lentils, chickpeas, black beans), oats, and vegetables (Brussels sprouts, carrots); insoluble fiber at 20–25 g/day from whole grains (quinoa, brown rice, whole wheat), vegetables (broccoli, leafy greens), and nuts and seeds; resistant starch at 15–30 g/day from cooled cooked potatoes, green bananas, cooked and cooled rice, and legumes. Overall target: 40–50 g total fiber daily from 30+ different plant species weekly, supporting maximal microbiome diversity.

*Fermented Foods Personalized to Microbiome Composition*: Baseline microbiome composition directs fermented food selection. Users with low *Lactobacillus* abundance (<2% relative abundance) receive priority recommendations for yogurt (6–8 oz daily, live cultures verified), kefir (6–8 oz daily), and sauerkraut (1/4 cup serving, 3–4 times weekly). Users with low *Bifidobacterium* abundance (<3%) receive emphasis on kimchi (1/4 cup daily), miso (1 tablespoon daily in soup or dressing), and tempeh (3–4 oz serving, 3–4 times weekly). Evidence from Wastyk et al. demonstrates six servings/day of fermented foods increased Shannon diversity +0.2 units and reduced IL-6 by 22% [11]. Platform target: 1–2 servings daily from diverse fermented sources, starting with 3–4 servings weekly for fermentation-naive users to allow for microbiome adaptation.

#### 4.4.2. Meal Timing

*Time-Restricted Eating*: A 10–12 h daily eating window aligned with circadian biology, personalized based on wearable sleep data and chronotype assessment. Most users receive recommendation for 8:00 AM–6:00 PM eating window, supporting circadian alignment and metabolic flexibility while remaining socially practical. Early chronotypes may shift window earlier (7:00 AM–5:00 PM) and late chronotypes slightly later (9:00 AM–7:00 PM).

*Caloric Distribution with Circadian Alignment*: Front-loading emphasis aligning with daily variation in insulin sensitivity: breakfast 30–35% of daily calories, lunch 40–45%, and dinner 20–25%. This pattern contrasts with typical Western pattern of small breakfast, moderate lunch, and large dinner and aligns with Blue Zone populations’ eating patterns.

*Hara Hachi Bu Principle*: Traditional Okinawan practice of eating to 80% fullness, supported through mindful eating prompts delivered via app 5 min into meals and visual portion guidance with hand-based measurements, with a typical result of 10–15% spontaneous caloric restriction without explicit calorie counting or restriction mentality.

#### 4.4.3. Evidence-Based Supplementation Protocols

Targeted supplementation when dietary sources prove insufficient or individual biomarker responses suggest benefit.

- **Omega-3 (EPA+DHA 2–3 g/day):** For elevated hs-CRP > 3 mg/L despite dietary modifications OR omega-3 index < 4%. Meta-analysis demonstrates approximately 18% hs-CRP reduction [19]. Target omega-3 index: 8–12%.
- **Curcumin (1000 mg with piperine):** For persistent inflammation (CRP > 3 OR IL-6 > 3 after 6-month interventions). Umbrella meta-analysis of 10 systematic reviews demonstrates clinically meaningful reductions in pro-inflammatory cytokines [20].
- **Multi-strain Probiotics:** For low microbiome diversity (Shannon < 3.0 despite dietary fermented foods). Includes *L. rhamnosus* GG (10^9^ CFU), *B. longum* (5 × 10^8^ CFU), *F. prausnitzii* (10^8^ CFU), and *L. plantarum* (5 × 10^8^ CFU).
- **Vitamin D3 (2000–4000 IU):** For deficiency (<30 ng/mL). Reduces hs-CRP in deficient individuals [21]. Target: 40–60 ng/mL.

All protocols include safety cross-referencing with user medications for drug–nutrient interaction checking.

Complete supplementation protocols with detailed dosages, indications, monitoring schedules, duration recommendations, and safety considerations are provided in Appendix F.

#### 4.4.4. Lifestyle Optimization

*Physical Activity*: Personalized targets are provided based on wearable baseline data, emphasizing consistent moderate activity over sporadic intense exercise. Target: 150–300 min moderate-intensity or 75–150 min vigorous-intensity activity weekly, plus strength training 2–3 times weekly for sarcopenia prevention. Minimum daily step target: 7000 steps. Meta-analyses demonstrate that 12–24-week exercise interventions reduce hs-CRP by 15–25% [22].

*Sleep Optimization*: Circadian alignment is based on HRV patterns and actigraphy data. Consistent 7–9 h nightly schedule is recommended with sleep–wake times varying <30 min day-to-day. Evening routine suggestions delivered 2 h before typical bedtime: blue light reduction (amber-filtering glasses or device night mode), bedroom temperature optimization (65–68 °F/18–20 °C), and progressive muscle relaxation or guided meditation. Poor sleep quantity (<6 or >9 h) and quality associate with 20–50% higher hs-CRP levels [22].

*Stress Management*: Personalized techniques are provided based on HRV patterns indicating autonomic nervous system balance and self-reported stress levels. Mindfulness-based stress reduction protocols with 10–20 min guided practices are delivered via app. Social connection emphasis is inspired by Blue Zone “moai” concept of lifelong social support networks. Evidence demonstrates HRV improvements correlate with hs-CRP reductions (r = −0.43, *p* < 0.01) [17].

### 4.5. User Interface Design

#### 4.5.1. Dashboard Visualization (Figure 3)

The primary dashboard provides at-a-glance inflammatory health status:

- Large L-II score display with color coding (green: 85–100, yellow: 70–84, orange: 55–69, red: <55).

**Figure 3:**
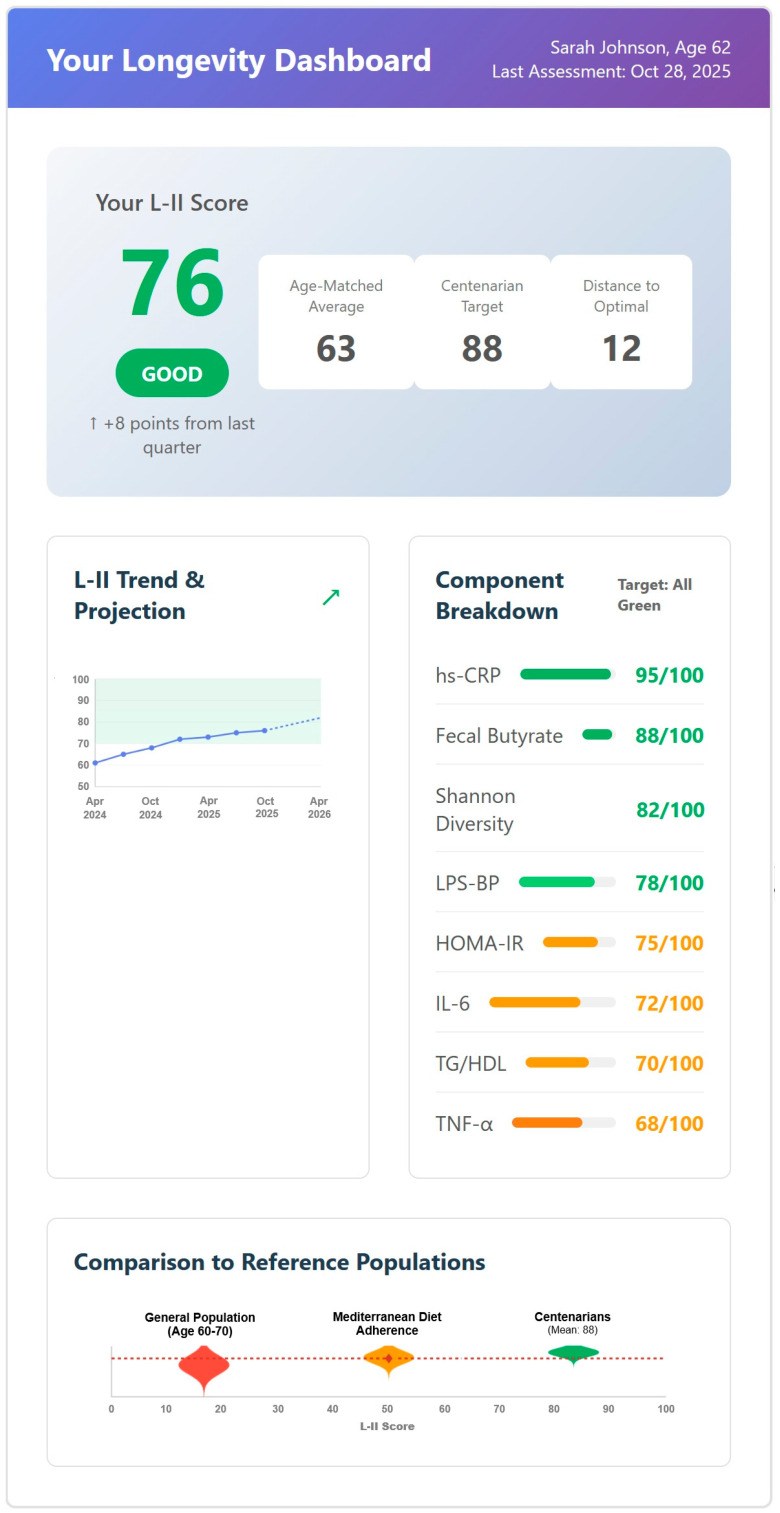
User dashboard interface for inflammation-centric health monitoring.

The dashboard provides intuitive visualization of the user’s inflammatory health status through four integrated panels designed following user-centered design principles and behavioral psychology research on health data visualization. *Main Score Card (top panel)* displays the current L-II composite score (76/100) with color-coded zone classification using universally recognized traffic light colors: green = “Good” (scores 70–84), yellow = “Moderate” (55–69), red = “Needs Attention” (<55), providing immediate at-a-glance status assessment. The quarterly change indicator (+8 points with upward arrow) highlights progress direction and magnitude, reinforcing positive behavior change through visible improvement feedback. Contextual comparisons provide three reference points anchoring the user’s achievement: (1) age-matched population average (63 for general population aged 60–70 years based on projected normative data), demonstrating user’s above-average status and providing social comparison motivation; (2) centenarian target profile (88 representing average L-II score from published centenarian cohorts), establishing aspirational goal and quantifying distance to optimal; (3) distance-to-optimal status explicitly stated (12 points to reach exceptional zone), converting abstract goal into concrete, achievable target. *Trend Graph (middle left panel)* shows historical L-II scores over the past 18 months with quarterly data points connected by a solid line, demonstrating progressive improvement trajectory from initial score of 61 (moderate zone, requiring intervention) to current 76 (good zone). The projected trajectory extends 6 months into the future (dashed line with 95% confidence interval shown as shaded region), calculated using ARIMA time-series forecasting based on current intervention adherence patterns. Annotations mark significant intervention initiation points (arrows with labels: “Started fermented food protocol” at month 6, “Increased plant diversity to 30+ species/week” at month 12), enabling users to connect specific behavior changes with subsequent biomarker improvements. *Component Breakdown (middle right panel)* displays individual biomarker performance using horizontal bar charts with intuitive left-to-right orientation and color coding matching score card zones. Green bars indicate components in optimal ranges requiring maintenance only: hs-CRP (0.8 mg/L, score 95/100—well below 1.0 mg/L optimal threshold), Shannon diversity (4.2, score 92/100—exceeding 3.8 centenarian threshold). Yellow bars show components in moderate status requiring continued attention: IL-6 (2.1 pg/mL, score 72/100), TNF-α (3.8 pg/mL, score 68/100). Components are ranked by improvement priority based on distance from optimal, component weight in L-II calculation, and responsiveness to available interventions. *Population Comparison (bottom panel)* contextualizes the user’s current L-II score relative to three reference populations using violin plots (kernel density plots showing distribution shape): (1) general population age 60–70 distribution (blue violin) shows mean of 63 ± 18 SD, with bimodal shape reflecting healthy vs. unhealthy subpopulations; (2) Mediterranean diet adherers (green violin) from published intervention studies shows mean of 78 ± 12 with narrower distribution and right-skewed shape; (3) centenarians (gold violin) representing exceptional longevity populations show mean of 88 ± 8 with very narrow distribution and normal shape. User’s current position (marked with prominent red diamond) demonstrates achievement above general population median (exceeding ~75th percentile), approaching Mediterranean diet cohort median (near 50th percentile of adherers), and achieving approximately 40–45% of the distance from the general population mean to centenarian mean. This inflammation-centric design differs fundamentally from conventional health apps by anchoring all visualizations to objective biological markers of longevity rather than weight, calories, or subjective wellness scores, providing users with transparent insight into their biological aging trajectory and evidence-based motivation for sustained intervention adherence.

- Trend graphs plotting L-II trajectory over 18–24 months with projected future path.
- Component breakdown showing which biomarkers need attention using horizontal bar charts.
- Population comparisons using violin plots contextualizing score relative to age-matched averages and centenarian targets.

#### 4.5.2. Personalized Action Feed

Recommendations delivered as 3–5 prioritized, actionable items refreshed daily based on current adherence patterns and upcoming opportunities:

- Daily food suggestions with specific recipes emphasizing anti-inflammatory ingredients matched to user’s dietary preferences and restrictions.
- Meal timing reminders aligned with user’s personalized eating window and circadian patterns.
- Activity prompts based on current step count and prolonged sedentary time detected by wearables.
- Sleep hygiene tips delivered 2 h before typical bedtime based on historical sleep data.

Each recommendation includes (1) a specific action to take with clear instructions, (2) the scientific rationale explained in accessible language without jargon, (3) the expected impact on L-II score based on predictive models, and (4) the difficulty level and time requirement for realistic planning.

#### 4.5.3. Progress Tracking and Behavioral Engagement

Behavioral psychology principles sustain long-term engagement through the following:

- Achievement milestones with digital badges for L-II improvements (5-point increments), dietary consistency streaks (7-day, 30-day, 90-day), and activity goals.
- Optional social features enabling community connection with users pursuing similar health goals, group challenges, and peer support.
- Personalized messaging adapting communication style to user preferences (educational vs. motivational emphasis) based on engagement pattern analysis.
- Celebration of biological improvements with positive reinforcement notifications (“Your hs-CRP dropped 25% this quarter! This improvement is associated with 18% reduced cardiovascular risk.”).

### 4.6. Clinical Integration

#### 4.6.1. Healthcare Provider Dashboard

The web-based interface facilitating integration with conventional healthcare workflows is detailed below.

*Population Management*: Overview of all enrolled patients with risk stratification based on L-II scores and trends, automated identification of patients with worsening inflammatory trajectories requiring clinical attention, population-level statistics tracking intervention effectiveness across entire patient panel, and comparative analytics showing clinic performance versus regional benchmarks.

*Individual Patient Tracking*: Comprehensive longitudinal view of inflammatory biomarkers, microbiome composition changes, and lifestyle metrics with graphical trend visualizations. Medication reconciliation system highlighting drugs that may influence inflammatory markers (NSAIDs, statins, corticosteroids, metformin). Clinical note integration with automated summary generation for inclusion in medical records. Secure HIPAA-compliant messaging enabling patient–provider communication about platform insights and recommendations.

*Clinical Decision Support*: Automated generation of clinical reports with biomarker trends and intervention recommendations formatted for medical record inclusion. Risk calculators integrating L-II components with traditional cardiovascular risk scores (Framingham, ASCVD) for comprehensive risk assessment. Referral recommendations when biomarkers suggest need for specialist evaluation (endocrinology for persistent metabolic dysregulation, gastroenterology for gut barrier dysfunction). Drug–nutrient interaction alerts for patients on medications with known interactions (warfarin with omega-3, statins with CoQ10 depletion).

#### 4.6.2. Compliance and Interoperability

HIPAA compliance maintained through end-to-end encryption, multi-factor authentication, role-based access controls with principle of least privilege, and comprehensive audit logging of all data access and modifications. ONC interoperability standards implementation using SMART on FHIR APIs enabling bidirectional EHR data exchange: patient demographics, problem lists, medication lists, laboratory results flowing from EHR to platform; platform-generated clinical summaries, trend reports, and recommendations flowing back to EHR. Providers retain ability to review and adjust algorithm-generated recommendations based on patient-specific considerations (comorbidities, allergies, contraindications, clinical judgment), with modifications immediately reflected in patient-facing mobile application, ensuring coherent care coordination.

### 4.7. Technical Implementation and Scalability

#### 4.7.1. Technology Stack

*Backend Infrastructure*: HIPAA-compliant cloud platform (AWS or Google Cloud Platform) with microservices architecture enabling modular development and independent scaling of system components. PostgreSQL for structured data (user profiles, biomarker results), MongoDB for semi-structured data (dietary logs, free-text notes), and Redis caching layer for real-time application responsiveness. Apache Kafka for event streaming from wearable devices enabling real-time activity monitoring, Apache Spark 3.5.0 for batch processing of computationally intensive microbiome sequencing data, custom Python 3.14.0/R 4.5.2 analytics pipeline for L-II calculation and statistical analysis, and TensorFlow 2.15.0/PyTorch 2.1.0 machine learning frameworks for predictive models.

*Mobile Applications*: Consumer-facing iOS and Android apps built with React Native framework for cross-platform code sharing and rapid development. On-device data caching enables offline functionality for dietary logging and activity tracking. Push notification system provides timely intervention delivery. HealthKit (iOS) and Google Fit (Android) integration enables automatic wearable data synchronization. MobileNetV2 computer vision module for food photo recognition. Native speech recognition APIs for voice-activated logging.

*Provider Dashboard*: Web-based application built with React.js frontend with responsive design supporting desktop and tablet devices. SMART on FHIR integration for EHR interoperability following ONC certification standards. Role-based access control supporting multiple care team member roles (physicians, nurses, dietitians, health coaches). Automated PDF report generation for medical record attachment and patient distribution.

#### 4.7.2. Security and Privacy Framework

*Data Protection*: AES-256 encryption at rest for all stored data in databases and file systems. TLS 1.3 encryption in transit for all client–server and server–server communications. End-to-end encryption for particularly sensitive biomarker results (genetic data, inflammatory markers) using client-side encryption keys. Hardware security modules (HSMs) in FIPS 140-2 Level 3 certified data centers for encryption key management.

*Access Controls*: Role-based permissions implementing principle of least privilege with granular access controls. Multi-factor authentication required for all user accounts (SMS, authenticator app, or biometric options). Session management with automatic timeout after 15 min of inactivity. Comprehensive audit logging of all data access, modifications, downloads, and sharing actions with tamper-proof log storage.

*Regulatory Compliance*: HIPAA compliance with signed Business Associate Agreements for all vendors handling protected health information. GDPR compliance for international users including data portability (machine-readable export), right to deletion (with appropriate retention for legal/regulatory requirements), and explicit consent mechanisms. Compliance with 21 CFR Part 11 for electronic records and signatures supporting regulatory submissions. Regular third-party security audits and penetration testing with annual SOC 2 Type II certification.

## 5. Supporting Evidence from Published Interventions (Streamlined)

While the platform represents novel integration, substantial published evidence supports its core components. This section synthesizes key findings validating the theoretical foundation.

The evidence is organized by L-II component and intervention type, with comprehensive synthesis provided in Table 2. Section 6.1 contextualizes these findings within the current digital health landscape.

### 5.1. Mediterranean Diet and Inflammatory Biomarkers

Multiple randomized controlled trials demonstrate that Mediterranean dietary patterns produce substantial reductions in systemic inflammatory markers. Casas et al. [10] (n = 164, 12 months) reported 32% hs-CRP reduction (3.5→2.4 mg/L, *p* < 0.001) and 18% IL-6 reduction (*p* = 0.03), occurring independently of weight loss and demonstrating direct anti-inflammatory effects. The landmark PREDIMED trial [12] (n = 7447, 4.8 years) demonstrated 30% cardiovascular event reduction (HR: 0.70, 95% CI: 0.54–0.92) with a Mediterranean diet supplemented with olive oil or nuts, with biomarker analyses revealing dose-dependent adherence–inflammation relationships: the highest adherence tertile showed hs-CRP 1.3 mg/L versus 2.4 mg/L in the lowest tertile (46% difference).

Ahmad et al. [3] established long-term clinical relevance in 25,994 participants followed for 25 years, demonstrating 23% reduced all-cause mortality (HR: 0.77, 95% CI: 0.73–0.83) with Mediterranean diet adherence. Pathway analyses revealed inflammatory biomarkers explained 10.2% and metabolic biomarkers explained 14.8% of the diet–mortality association, establishing both short-term biomarker improvements and long-term outcomes. Shannon et al.’s [28] meta-analysis (27 RCTs, n = 2163) demonstrated significant flow-mediated dilation improvements (SMD: 0.40, 95% CI: 0.18–0.63, *p* < 0.001), with effects correlating with inflammatory marker reductions (r = −0.52, *p* < 0.001), suggesting a mechanistic linkage between dietary pattern, inflammation, and vascular health.

### 5.2. Microbiome-Targeted Interventions

Wastyk et al. [11] (n = 36, 17 weeks) demonstrated that fermented food intervention (six servings/day) increased microbiome diversity (Shannon +0.2 units, 3.41 → 3.61, *p* = 0.01) and reduced IL-6 by 22% (*p* = 0.04), with effects persisting 4 weeks post-intervention. Diversity increases inversely correlated with baseline diversity (r = −0.61, *p* < 0.001), indicating the greatest benefit for individuals with lower initial diversity—a population identifiable through baseline microbiome assessment. Centenarian microbiome studies provide mechanistic support: Pang et al. [2] (n = 297) found a 1.4-fold enrichment in butyrate synthesis genes with 45% higher fecal butyrate (18.2 ± 4.3 vs. 12.6 ± 3.8 μmol/g, *p* < 0.001), while Biagi et al. [15] (n = 24 Italian centenarians) documented distinctive signatures including increased diversity (Shannon 3.92 ± 0.48 vs. 3.31 ± 0.52, *p* < 0.001) and *Akkermansia* enrichment (4.8% vs. 1.2%, *p* < 0.001).

Fiber diversity effects are demonstrated by Menni et al. [29] (n = 1103, TwinsUK cohort), showing that high fiber intake (>30 g/day) and microbiome diversity independently predicted lower metabolic syndrome prevalence (OR: 0.32 and 0.41, respectively), with high-fiber maintainers showing 42% lower weight gain over 9.5 years (*p* < 0.001). Meslier et al. [24] (n = 82, 8 weeks) provided mechanistic insight: Mediterranean diet increased butyrate-producing taxa (*F. prausnitzii* +37%, *Roseburia* +29%, both *p* < 0.01) with a corresponding 18% butyrate increase (*p* = 0.02), occurring independently of weight loss and correlating with cholesterol and hs-CRP improvements.

The ISAPP consensus statement on fermented foods [30] synthesized evidence concluding that regular consumption associates with improved digestive health, enhanced immune function, and potential inflammatory marker reduction, with effects mediated through viable microbial delivery and bioactive metabolite production, providing scientific rationale for personalized fermented food recommendations based on individual microbiome composition.

### 5.3. Digital Health Engagement Feasibility

Meta-analytic evidence demonstrates feasible long-term engagement with digital health nutrition interventions. Lyzwinski et al. [26] analyzed 48 platforms, reporting 12-month retention rates of 58–74% (intensive interventions up to 84%) with dietary logging averaging 4.2–6.1 days/week among active users. Sustained engagement predictors included personalized feedback (HR: 0.42 for disengagement), wearable integration (+18% retention), educational content (+12%), and social features (+15%)—all core platform features. Ma et al.’s [31] pilot cohort study demonstrated that older adults (mean age 75 years) maintained wearable device adherence rates of 86.9–98.4% (median 92.1%) during monitoring periods extending over multiple months, dispelling technology adoption concerns in this demographic.

McConnell et al. [32] (n = 50,000, MyHeart Counts) successfully integrated Apple Watch data with periodic surveys, with 40% providing continuous data over 12 months. Participants receiving personalized feedback showed 2.3× longer engagement (median 8.2 vs. 3.6 months, *p* < 0.001), supporting the value of data-driven recommendations and establishing a proof of concept for large-scale digital health data integration with objective health metrics.

### 5.4. Multi-Component Integration Evidence

#### NU-AGE Trial

The NU-AGE trial demonstrates benefits of personalized multi-component interventions. Berendsen et al. [33] (n = 1296, 12 months, five countries) reported that personalized Mediterranean counseling—individualized assessments, culturally adapted recommendations, tailored recipes, key food provision, regular feedback—achieved a Mediterranean Diet Adherence Score increase of +2.8 points versus +0.6 for generic advice (between-group difference: 2.2, 95% CI: 1.8–2.6, *p* < 0.001), demonstrating personalization drives adherence despite similar contact intensity.

Santoro et al.’s [23] biomarker analyses showed that high adherers (MEDAS ≥ 9) achieved hs-CRP reductions of 18% (2.8 → 2.3 mg/L, *p* = 0.02) and IL-6 reductions of 12% (2.9 → 2.6 pg/mL, *p* = 0.04). The greatest improvements occurred in the poorest baseline adherers (hs-CRP −28% in MEDAS < 5 vs. −8% in MEDAS ≥ 9, interaction *p* = 0.01) and in those with the highest baseline inflammation (hs-CRP −34% in CRP > 3 mg/L vs. −11% in CRP < 1 mg/L, interaction *p* = 0.003), demonstrating the “greatest benefit for those needing it most”—individuals identifiable through the platform’s baseline L-II assessment.

Table 2 synthesizes these published findings supporting each L-II component’s responsiveness to interventions, digital platform engagement feasibility, and long-term clinical outcomes. This evidence demonstrates that while individual platform components are well-validated through published RCTs and meta-analyses, the integrated system itself represents a novel contribution requiring prospective validation.

This evidence demonstrates that while individual platform components are well-validated through published RCTs and meta-analyses, the integrated system itself represents a novel contribution requiring prospective validation.

### 5.5. Cost-Effectiveness Evidence

Dalziel et al.’s [27] evaluation in vascular disease patients demonstrates the following: Nutrition interventions demonstrated ICERs of USD 2100–4800/QALY—well below the USD 50,000/QALY threshold. Net healthcare savings of USD 890–1450/patient/year through reduced hospitalizations (22% reduction, *p* = 0.02), emergency visits (31% reduction, *p* = 0.01), and medication costs (12% reduction, *p* = 0.04).

PREDIMED economic modeling indicates the following: Mediterranean diet in high-risk populations showed a cost-saving profile with estimated savings of EUR 1040 per participant over 5 years when accounting for prevented cardiovascular events.

### 5.6. Evidence Gap Analysis

Published evidence validates individual components but highlights critical gaps that the integrated platform addresses. These gaps represent opportunities that the integrated platform addresses, as discussed further in Section 6.3 regarding limitations and in Section 6.4 regarding validation priorities.

**Monitoring Frequency:** Most trials assess biomarkers only at baseline and endpoint, missing dynamic trajectories. The platform’s quarterly assessments enable early responder detection and intervention adjustment.

**Personalization:** Trials apply uniform protocols despite heterogeneous responses. NU-AGE demonstrated the greatest improvements in poorest baseline adherers and those with the highest inflammation, suggesting benefits from targeting. The platform’s AI adapts to genetic variants, microbiome composition, and observed responses.

**Integration:** Existing platforms rarely integrate objective biological measures with continuous lifestyle data. The platform’s L-II synthesizes multi-omic data into actionable metrics.

**Sustainability:** Meta-analyses show that adherence typically peaks during interventions and declines thereafter (only 30–40% maintain changes at 12 months). The platform’s continuous feedback loop—where users observe biological responses—aims to enhance intrinsic motivation.

**Mechanistic Understanding:** The platform’s integrated data collection—correlating specific foods with microbiome changes and inflammatory trajectories—could help elucidate mechanistic pathways through within-person longitudinal analyses.

## 6. Discussion

### 6.1. Platform Positioning in Digital Health Landscape

The proposed platform addresses critical gaps in existing digital nutrition applications. Current platforms like MyFitnessPal, Noom, and ZOE focus primarily on calorie tracking or weight management, with none integrating objective inflammatory biomarkers with continuous lifestyle monitoring to target biological aging mechanisms.

#### Key Precedents

- **ZOE Predict Studies:** Integrated microbiome sequencing with continuous glucose monitoring for personalized nutrition; focuses on metabolic health; **lacks inflammatory biomarkers as primary outcomes.**
- **InsideTracker:** Biomarker-driven recommendations; focuses on individual nutrients rather than dietary patterns; **lacks microbiome integration or continuous lifestyle monitoring.**
- **Viome:** Microbiome-based dietary recommendations; **does not integrate inflammatory markers or validate through longitudinal biomarker tracking**, limiting ability to assess intervention effectiveness.
- **Traditional dietitian counseling:** Evidence-based dietary guidance; **lacks continuous monitoring, objective biomarker validation, and scalability** to large populations.

**Unique Value Proposition:** The platform uniquely combines inflammatory biomarker validation (quarterly L-II assessments) with microbiome-guided personalization and continuous lifestyle tracking—integration not present in existing systems. By anchoring recommendations to objective inflammatory status rather than subjective goals, the platform shifts focus from weight management to healthspan optimization.

Building on these precedents while addressing identified gaps, the proposed platform offers unique value propositions, as detailed in Section 6.2.

### 6.2. Strengths and Economic Viability

**Evidence-Based Foundation:** All platform components derive from published RCTs demonstrating efficacy. Mediterranean diet interventions show consistent 18–32% hs-CRP reductions [10], microbiome-targeted diets increase diversity 6–28% [11], and digital platforms achieve 58–84% retention at 12 months [26].

**Biological Objectivity:** The L-II tracks objective inflammatory status—the mechanistic driver of biological aging—providing users with transparent insight into their biological aging trajectory rather than appearance-based metrics.

**Personalization Potential:** AI-driven adaptation to individual genetics, microbiome composition, and observed biomarker responses enable precision nutrition approaches. The Random Forest model (R^2^ = 0.58 for L-II prediction) provides personalized recommendations with quantified outcomes and confidence intervals.

**Scalability:** Digital delivery with quarterly mail-in biomarker collection enables broad reach compared to intensive in-person interventions. The modular architecture supports tiered service models: basic (dietary tracking), standard (quarterly L-II), and premium (genetic testing).

**Economic Viability:** In modeled scenarios, economic projections suggest an ICER of approximately USD 1667/QALY—well below the USD 50,000 threshold. Sensitivity analyses demonstrate robustness: varying effectiveness (20–50% event reduction: ICER USD 890–4200/QALY) varying costs (USD 300–600/year: ICER USD 1100–3800/QALY). Healthcare savings are projected at USD 1240/year through reduced hospitalizations and emergency visits. For employers, a net ROI of USD 960–1780 per participating employee annually is estimated. These projections are based on assumptions and require confirmation in real-world implementation studies. Multiple reimbursement pathways exist: chronic care management codes (USD 42–65/month), remote therapeutic monitoring (new CPT codes 2022), value-based payment arrangements, and employer wellness programs.

**Critical Caveat:** All projections require validation through actual implementation data and real-world cost tracking.

### 6.3. Critical Appraisal and Limitations

Despite the strengths outlined in Section 6.2, several critical limitations must be acknowledged and addressed before clinical implementation. These limitations span scientific validity, implementation feasibility, and ethical considerations.

**Unproven Integration Hypothesis:** While individual components show efficacy, whether the integrated approach produces synergistic benefits exceeding the sum of its parts remains an empirical question requiring RCT validation. Integration may add complexity without proportional benefit.

**Biomarker Variability:** Inflammatory markers exhibit 20–40% intra-individual coefficient of variation. Quarterly assessments may capture measurement noise rather than true biological change, necessitating sophisticated statistical approaches to distinguish signal from noise.

**Generalizability Concerns:** Published Mediterranean diet trials enrolled predominantly white, educated participants from Southern Europe or North America [10,12,23,33]. The platform’s effectiveness in diverse, real-world populations with varying socioeconomic status, health literacy, and technology access requires demonstration.

**AI Interpretability:** Complex machine learning models may generate recommendations that are difficult to explain to users and clinicians, potentially limiting trust and adoption despite accuracy. Mitigation strategies: simplified explanation interfaces (“top 3 factors driving this recommendation”), SHAP values quantifying each feature’s contribution, example cases showing similar users’ responses, provider override capability.

**Cost and Accessibility Barriers:** The USD 350–450/year subscription cost (representing 0.5–0.6% of median household income, 1.2–1.5% for <USD 30 K/year households) may be prohibitive for substantial portions of the population. Platform requirements (smartphone ownership 85% overall but lower in older adults 65+ [73%], low-income <USD 30 K [76%], rural residents) create additional access barriers. Essential equity strategies: sliding-scale pricing with income-based subsidies, community health center partnerships serving underserved populations, multi-language support with culturally adapted recommendations, simplified interface versions for limited digital literacy, device lending programs.

**Wearable Data Validity:** Consumer wearables show limitations in accuracy. Technical specifications were moved to Appendix D to maintain narrative flow. Summary: step count 85–95% accurate during normal walking (lower during slow walking or non-ambulatory activities), sleep monitoring 65–75% agreement with polysomnography, HRV correlation r = 0.85–0.92 at rest (lower during activity). Platform emphasizes higher-accuracy metrics while de-emphasizing less reliable measures.

**Adherence Sustainability:** Realistic projections show declining engagement: dietary logging decreases from 5–6 to 2–3 days/week by month 6 [26], biomarker collection compliance declines from 85–92% (year 1) to 70–75% (year 2), and only 30–40% maintain substantial intervention changes at 12 months [23,31,32,33]. **Mitigation strategies:** Achievement milestones with badges, social features enabling community connection, personalized messaging adapted to user preferences, celebration of biological improvements providing positive reinforcement. However, even optimized strategies will not achieve universal sustained adherence.

#### Ethical Considerations

*Data Privacy*: The platform handles sensitive genetic information (five SNPs), complete microbiome composition, inflammatory biomarker profiles, and longitudinal lifestyle data. Privacy risks include re-identification potential, genetic discrimination (GINA does not cover life insurance), health/employer discrimination, data breach impacts, and secondary use concerns. Implemented protections include AES-256 encryption, HIPAA/GDPR compliance, multi-factor authentication, and audit logging. *Complete privacy specifications are given in Appendix E*. Unresolved challenges remain regarding long-term data governance and evolving regulatory landscape.

*Equity and Access*: As detailed above, the platform must address health equity through concrete strategies, ensuring diverse populations have access and derive comparable benefits.

*Algorithmic Fairness*: AI systems risk perpetuating biases through training data predominantly from white, educated populations; outcome definition bias; feature selection bias; and disparate impact across demographic groups. Mitigation: diverse training datasets, regular bias audits across demographic groups, transparency mechanisms, human oversight, and appeals processes.

**Technical Feasibility:** The platform requires HIPAA-compliant cloud infrastructure with microservices architecture, mobile applications with offline functionality, provider dashboards with EHR interoperability via SMART on FHIR. Development costs: USD 1.25–2.05 M initial. Ongoing per-user costs: USD 180–260/year. *Complete technical specifications are given in Appendix D*.

**Implementation Barriers:** Healthcare system adoption faces provider time constraints, reimbursement uncertainty, medical liability concerns, and workflow resistance. User engagement challenges include information overload, health literacy barriers, privacy concerns, and motivational heterogeneity.

### 6.4. Future Directions and Validation Priorities

Addressing the limitations detailed in Section 6.3 requires a systematic validation roadmap progressing from proof-of-concept studies to large-scale implementation trials.

#### 6.4.1. Critical Validation Questions

1. **Does integration produce synergistic benefits?** A four-arm RCT comparing full platform vs. dietary education alone vs. digital tracking without biomarkers vs. usual care would establish incremental benefit.
2. **What is the optimal assessment frequency?** Trials comparing monthly, quarterly, and biannual biomarker schedules would determine the cost–benefit balance.
3. **How does personalization improve outcomes?** Factorial design randomizing to personalized vs. population-average recommendations would isolate value-added.
4. **What populations benefit most?** Subgroup analyses would establish whether the platform provides universal benefit or should be targeted to high-risk individuals with elevated baseline inflammation.
5. **What is durability of effects?** Long-term follow-up (3–5 years) would determine whether quarterly biomarker feedback sustains behavior change and engagement over years required for chronic disease prevention.

#### 6.4.2. Proposed Validation Roadmap

*Phase 2 Multi-Center Effectiveness Trial*: RCT with 500 participants across five sites over 24 months. Primary outcome: change in L-II score. Secondary outcomes: cardiovascular events, diabetes incidence, healthcare utilization, QALYs, cost-effectiveness. Sample size provides 90% power to detect 10-point L-II difference and 30% event reduction.

*Phase 3 Real-World Implementation*: Pragmatic trial in 10 healthcare systems with 2000 patients and 36-month follow-up. Assessment of adoption rates, sustained engagement, clinical workflow integration, and health outcomes including major adverse cardiovascular events, all-cause mortality, and healthcare costs.

#### 6.4.3. Technology Enhancement Roadmap

*Near-term (0–12 months)*: Biomarker expansion (AGEs, oxidized LDL, expanded cytokines), AI improvements (training dataset expansion to 1000+ users, deep learning for food recognition >90% accuracy, NLP for dietary habit extraction).

*Medium-term (1–3 years)*: Advanced monitoring integration (continuous glucose monitors, breath ketone monitoring), microbiome enhancements (shotgun metagenomics standard, virome/mycobiome characterization, expanded metabolomics beyond SCFAs).

*Long-term (3–5 years)*: Wearable biosensors for continuous inflammatory tracking, pharmacogenomics integration for drug–nutrient interactions, epigenetic profiling, proteomics/lipidomics, single-cell immune profiling.

**Research Priorities:** Critical mechanistic studies addressing how rapidly dietary changes alter inflammatory biomarkers (time-course studies with weekly sampling), which microbiome changes causally drive inflammation reduction (fecal microbiota transplantation studies), whether genetic variants modify dietary intervention responses (nutrigenomics substudies with interaction analyses), and what role circadian factors play (chronobiology studies comparing early versus late eating patterns). Population-specific studies determining whether L-II thresholds differ across ethnic groups, how the platform performs in younger adults (30–45 years), whether the approach benefits patients with established inflammatory diseases, and optimal intervention intensity for different risk levels. Comparative effectiveness research comparing platform versus traditional dietitian counseling versus anti-inflammatory medications in metabolic syndrome, various dietary patterns (Mediterranean versus Blue Zone-specific), and digital-only versus digital plus human coaching.

## 7. Conclusions

Chronic low-grade inflammation represents a central, modifiable mechanism of biological aging that responds to dietary and lifestyle interventions. This paper proposes a comprehensive digital health platform integrating quarterly inflammation and microbiome assessments with continuous lifestyle monitoring to deliver personalized longevity interventions based on Mediterranean and Blue Zone dietary principles. The Longevity-Inflammation Index (L-II) provides an actionable composite biomarker synthesizing eight components—inflammatory markers (hs-CRP, IL-6, TNF-α), microbiome indicators (Shannon diversity, butyrate production, LPS-binding protein), and metabolic parameters (HOMA-IR, TG/HDL ratio)—into a single score tracking inflammatory status and enabling objective assessment of intervention responses. This inflammation-centered approach differs fundamentally from existing digital health platforms by anchoring all recommendations to objective biological markers validated quarterly rather than subjective goals or generic dietary guidelines.

Published evidence from multiple intervention studies supports the platform’s feasibility and expected efficacy. Mediterranean dietary interventions demonstrate 18–32% reductions in hs-CRP [10], microbiome-targeted interventions achieve 6–28% increases in diversity [11], and long-term adherence associates with 23% reduced all-cause mortality [3]. Digital health platforms demonstrate sustained engagement rates of 58–84% at 12 months [26], with wearable device adherence of 75–92% in older adults [31]. In modeled scenarios, economic projections suggest favorable cost-effectiveness ratios (approximately USD 1667/QALY) that are well below standard thresholds, with potential healthcare savings of USD 890–1450 per patient annually [27]. These projections are based on assumptions and require confirmation in real-world implementation studies. These findings establish that the proposed platform’s individual components are evidence-based and achievable.

However, the integrated system requires rigorous validation through prospective trials to establish whether multi-component integration produces outcomes exceeding individual components. Critical limitations include the unproven integration hypothesis, biomarker variability challenges (20–40% intra-individual CV), generalizability concerns requiring validation in diverse populations, cost and accessibility barriers necessitating equity strategies, and adherence sustainability challenges, given that only 30–40% maintain dietary changes at 12 months in published trials [23,33]. The proposed validation roadmap addresses these limitations through Phase 2 multi-center effectiveness trials (n = 500, 24 months), establishing biomarker efficacy, and Phase 3 real-world implementation studies (n = 2000, 36 months), demonstrating clinical outcomes, sustained engagement, and economic viability across diverse healthcare systems and patient populations.

As validation studies progress and technology advances, inflammation-centered digital health interventions may become standard components of preventive healthcare, democratizing access to personalized longevity strategies derived from the world’s longest-lived populations. The platform’s modular architecture enables continuous refinement through expanding biomarker panels (advanced glycation end-products, oxidized LDL, expanded cytokines), enhancing AI prediction algorithms as real-world data accumulates, and integrating emerging technologies including continuous inflammatory biosensors and pharmacogenomic testing. Success will ultimately be measured not merely by biological improvements but by meaningful extension of healthspan—years lived in good health—across socioeconomically and ethnically diverse populations worldwide. By continuously monitoring inflammatory status and delivering evidence-based interventions adapted to individual biology, this framework represents a paradigm shift from reactive disease treatment to proactive optimization of the biological aging process itself.

## Figures and Tables

**Table 1:** Longevity-Inflammation Index (L-II) components and scoring criteria.

Component	Weight	Optimal Target	Moderate Range	High Risk	Scoring Algorithm	Measurement Method
hs-CRP (mg/L)	20%	<1.0	1.0–3.0	>3.0	100 pts at <1.0; linear decrease to 0 at >5.0	Immunoturbidimetry from finger-stick blood
IL-6 (pg/mL)	15%	<1.5	1.5–3.0	>3.0	100 pts at <1.5; linear decrease to 0 at >6.0	ELISA from finger-stick blood
TNF-α (pg/mL)	15%	<3.0	3.0–6.0	>6.0	100 pts at <3.0; linear decrease to 0 at >10.0	ELISA from finger-stick blood
Fecal butyrate (μmol/g)	10%	>15	10–15	<10	100 pts at >20; linear decrease to 0 at <5.0	GC-MS from stool
Shannon diversity	10%	>3.8	3.0–3.8	<3.0	100 pts at >4.5; linear decrease to 0 at <2.0	16S rRNA sequencing from stool
LPS-binding protein (μg/mL)	10%	<20	20–30	>30	100 pts at <20; linear decrease to 0 at >50	ELISA from finger-stick blood
HOMA-IR	10%	<2.0	2.0–3.0	>3.0	100 pts at <2.0; linear decrease to 0 at >5.0	Calculated from fasting glucose/insulin
TG/HDL ratio	10%	<2.0	2.0–3.0	>3.0	100 pts at <1.5; linear decrease to 0 at >5.0	Calculated from lipid panel

Note: Scoring algorithms are linear between specified points. Final L-II score = Σ(Component Score × Component Weight). Optimal targets derive from centenarian population means [2,5,6]; high-risk thresholds derive from cardiovascular outcome studies [7,8].

**Table 2:** Published evidence supporting platform component Efficacy.

L-II Component	Study	Design	Primary Findings	Relevance
hs-CRP	Casas 2014 [10]	RCT, Med diet, 12 months, n = 164	hs-CRP: −32% (3.5→2.4 mg/L, *p* < 0.001)	Validates responsiveness; supports 20% weighting
IL-6	Wastyk 2021 [11]	RCT, fermented foods, 17 weeks, n = 36	IL-6: −22% (*p* = 0.04); 19 markers assessed	Shows microbiome-targeted inflammation reduction
TNF-α	Santoro 2018 [23]	NU-AGE analysis, 12 months, n = 1296	High vs. low adherers: TNF-α: −15% (*p* = 0.03)	Validates responsiveness in older adults
Shannon Diversity	Wastyk 2021 [11]	RCT, 17 weeks, n = 36	Shannon: +0.2 units (3.41→3.61, *p* = 0.01)	Demonstrates dietary modifiability
Fecal Butyrate	Meslier 2020 [24]	Controlled feeding, 8 weeks, n = 82	Butyrate: +18% (*p* = 0.02); taxa: +37%	Establishes Med diet effects on production
LPS-BP	Seethaler 2022 [25]	Intervention, 8 weeks, n = 44	LPS-BP: −19% (*p* = 0.04); Zonulin: −25%	Validates gut barrier improvements
HOMA-IR	PREDIMED 2018 [12]	RCT, 4.8 years, n = 7447	HOMA-IR: −22% (calculated)	Demonstrates metabolic improvements
TG/HDL	PREDIMED 2018 [12]	RCT, 4.8 years, n = 7447	TG/HDL: −18% in EVOO group (*p* < 0.001)	Establishes lipid ratio improvements
Digital Engagement	Lyzwinski 2019 [26]	Meta-analysis, 48 studies	12 mo retention: 58–84%; Logging: 4–6 d/wk	Validates sustained engagement feasibility
Long-term Outcomes	Ahmad 2024 [3]	Cohort, 25 years, n = 25,994	Mortality HR: 0.77 (95% CI: 0.73–0.83)	Establishes clinical relevance
Cost-Effectiveness	Dalziel 2017 [27]	Economic analysis	ICER: USD 2100–4800/QALY; Savings: USD 890–1450/pt/yr	Supports economic viability

Abbreviations: RCT = randomized controlled trial; Med = Mediterranean; EVOO = extra-virgin olive oil; HR = hazard ratio; ICER = incremental cost-effectiveness ratio; QALY = quality-adjusted life year; LPS-BP = LPS-binding protein.

## Data Availability

This manuscript presents a conceptual framework based on the published literature. No original data were collected. All cited evidence is available through the referenced published sources and their respective data repositories.

## Appendix A. Detailed L-II Calculation Examples

Complete worked examples showing conversion of individual biomarker values to component scores, weighting, and summation to final L-II score across all five interpretation zones (Exceptional, Good, Moderate, Suboptimal, High Risk).

## Appendix B. AI Model Technical Specifications

Complete Random Forest hyperparameters (500 trees, max depth 15, min samples per leaf 10, √45 ≈ 7 features per split, bootstrap sampling enabled). Detailed list of 45 input features with categories: 8 baseline L-II components, 5 genetic variants (FOXO3 rs2802292, IL-6 rs1800795, TNF-α rs1800629, APOE ε4, CRP rs1205), 15 key microbiome taxa (*Akkermansia*, *Faecalibacterium*, *Lactobacillus*, *Bifidobacterium*, *Roseburia*, *Eubacterium*, *Ruminococcus*, *Prevotella*, *Bacteroides*, *Christensenellaceae*, *Verrucomicrobia*, *Firmicutes ratio*, *Proteobacteria*, *Actinobacteria*, butyrate-producing taxa composite), 12 dietary variables (Mediterranean Diet Adherence Score, Dietary Inflammation Index, fiber intake g/day, polyphenol intake mg/day, omega-3:omega-6 ratio, plant species diversity count/week, fermented food servings/week, processed food frequency, added sugar g/day, red meat servings/week, whole grain servings/day, meal timing regularity), 5 demographic/lifestyle factors (age, sex, BMI, physical activity minutes/week, sleep hours/night). Cross-validation procedures (5-fold stratified repeated 10 times). Feature importance rankings with top 10 predictors. Calibration curves showing predicted vs. observed L-II changes across deciles.

## Appendix C. Economic Model Assumptions and Sensitivity Analyses

Base-case assumptions: intervention cost USD 450/year, comparator USD 50/year, 10-year horizon, 3% discount rate, healthcare system perspective, 35% CV event reduction, 28% diabetes prevention, CV event costs (USD 25,000 per MI, USD 30,000 per stroke), diabetes annual costs (USD 9600). Sensitivity analysis results varying intervention effectiveness (20–50% event reduction: ICER USD 890–4200/QALY), costs (USD 300–600/year: ICER USD 1100–3800/QALY), time horizon (5–15 years: ICER USD 4100-USD 980/QALY), discount rate (0–5%: ICER USD 1200–2400/QALY), baseline CV risk (low–high: ICER USD 3200-USD 980/QALY), adherence rate (40–80%: ICER USD 2800-USD 1200/QALY). Probabilistic sensitivity analysis Monte Carlo simulation (10,000 iterations) demonstrating 78% probability ICER <USD 50,000/QALY and 92% probability ICER <USD 100,000/QALY.

## Appendix D. Clinical Integration Protocols

Provider onboarding workflow (initial training webinar 60 min, sandbox environment access, clinical champion designation, go-live support, monthly office hours). Patient enrollment procedures (eligibility identification, shared decision-making discussion, informed consent, baseline biomarker kit mailing, app download, onboarding call with health coach). Alert thresholds triggering provider notifications (hs-CRP >10 mg/L, new onset hyperglycemia, sustained L-II decline >15 points over two quarters, patient-reported symptoms, adherence concerns). Documentation templates for EHR integration. Technical specifications: HIPAA-compliant cloud infrastructure, microservices architecture, mobile app features, provider dashboard capabilities, security measures, interoperability standards. Wearable device accuracy specifications: step count 85–95% accuracy normal walking (lower during slow walking <2 mph [~70%], ADLs [~60%], upper-body exercises [<50%]), sleep monitoring 65–75% agreement with polysomnography, HRV correlation r = 0.85–0.92 at rest (r = 0.60–0.75 moderate exercise, r = 0.40–0.60 vigorous).

## Appendix E. Privacy and Security Technical Specifications

Encryption standards (AES-256 at rest, TLS 1.3 in transit, end-to-end for genetic/biomarker results, HSMs for key management in FIPS 140-2 Level 3 certified data centers). Access control matrices defining permissions by role (Patient, Provider, Health Coach, Administrator, Developer). Audit logging specifications (all data access logged with timestamp, user ID, IP address, action performed; logs retained 7 years per HIPAA; anomaly detection algorithms; quarterly audit log reviews with annual third-party audits). HIPAA/GDPR compliance procedures (Business Associate Agreements, Data Processing Agreements, annual HIPAA Security Risk Assessments, breach notification procedures meeting 72 h GDPR requirement, data portability tools, right to deletion implementation with 30-day turnaround). Detailed discussion of genetic discrimination risks (GINA limitations not covering life insurance), data breach impact scenarios, long-term data governance challenges.

## Appendix F. Supplementation Protocols and Evidence

**Omega-3 Fatty Acids (EPA+DHA):** Full protocol including indication (hs-CRP > 3 mg/L despite 3 months of dietary modifications OR omega-3 index <4% OR inadequate dietary fish intake <1 serving/week), dosage (2–3 g/day combined EPA+DHA from molecularly distilled fish oil third-party tested for mercury/PCBs or algal oil for vegetarians), evidence (meta-analysis of randomized controlled trials demonstrating weighted mean difference −0.20 mg/L [95% CI: −0.28 to −0.12], representing approximately 18% CRP reduction in patients with chronic non-autoimmune diseases [19]), monitoring (omega-3 index via dried blood spot at baseline and 6 months, target 8–12%), duration (minimum 3–4 months for RBC membrane incorporation and maximal anti-inflammatory effects), safety (contraindications: fish/seafood allergy, concurrent anticoagulant therapy warfarin/rivaroxaban due to bleeding risk, upcoming surgery within 2 weeks; side effects: fishy aftertaste, mild GI upset mitigated by taking with meals).

**Curcumin:** Indication (persistent inflammatory elevation hs-CRP > 3 mg/L OR IL-6 > 3 pg/mL after 6 months dietary/lifestyle interventions), dosage (1000 mg curcumin extract standardized to 95% curcuminoids combined with 5–10 mg piperine enhancing absorption 20–40× or formulated with phosphatidylcholine, divided doses 500 mg twice daily with meals), evidence (randomized controlled trial in metabolic syndrome patients demonstrating statistically significant reductions in TNF-α and IL-6 versus placebo [*p* < 0.001] with bioavailable formulation [20]; multiple meta-analyses support anti-inflammatory effects across diverse populations), duration (8–12-week trial with biomarker reassessment, discontinue if no improvement after 12 weeks), safety (contraindications: gallbladder disease, concurrent antiplatelet agents aspirin/clopidogrel due to bleeding risk; side effects: mild GI upset, yellow stool harmless).

**Multi-Strain Probiotics:** Indication (low microbiome diversity Shannon <3.0 despite 3 months dietary fermented food intake 1+ servings daily OR specific taxa deficiencies on baseline testing), formulation (*L. rhamnosus* GG 10^9^ CFU, *B. longum* 5 × 10^8^ CFU, *F. prausnitzii* 10^8^ CFU if available [limited commercial availability], *L. plantarum* 5 × 10^8^ CFU), evidence (RCT evidence from Wastyk et al. [11] demonstrating microbiome diversity increases and inflammatory marker reductions with fermented food intervention; probiotic supplementation shows similar but typically smaller effects than whole-food sources), monitoring (microbiome reassessment at 3 months evaluating taxa abundance changes and Shannon diversity improvement), duration (minimum 3 months for stable microbiome integration, consider maintenance at lower dose if beneficial effects demonstrated), safety (contraindications: severely immunocompromised patients, central venous catheters due to rare bacterial translocation risk; side effects: initial bloating/gas typically resolving within 1–2 weeks as microbiome adapts).

**Vitamin D3:** Indication (baseline 25-OH vitamin D <30 ng/mL, prevalent in 40%+ of general population, higher in winter months, limited sun exposure, darker skin pigmentation, obesity), dosage (2000–4000 IU [50–100 mcg] daily based on baseline level, BMI, sun exposure; lower baseline <20 ng/mL requires higher dosing), evidence (vitamin D deficiency associates with elevated inflammatory markers; supplementation in deficient individuals reduces hs-CRP meta-analysis mean reduction 0.5 mg/L in deficient individuals [21]), monitoring (retest 25-OH vitamin D at 3–4 months to assess repletion and adjust dose, target 40–60 ng/mL consensus optimal range balancing bone health, immune function, avoiding toxicity), form (vitamin D3 cholecalciferol preferred over D2 ergocalciferol due to superior bioavailability and longer duration of action), safety (well-tolerated at recommended doses; vitamin D toxicity rare with doses <10,000 IU/day; monitor calcium levels if taking high doses >4000 IU/day long-term).
